# Multidrug resistance in chronic myeloid leukaemia: how much can we learn from MDR–CML cell lines?

**DOI:** 10.1042/BSR20130067

**Published:** 2013-11-25

**Authors:** Vivian M. Rumjanek, Raphael S. Vidal, Raquel C. Maia

**Affiliations:** *Instituto de Bioquímica Médica, Universidade Federal do Rio de Janeiro, Rio de Janeiro, Brazil; †Programa de Pesquisa em Hemato-Oncologia Molecular, Instituto Nacional de Câncer (INCA), Rio de Janeiro, Brazil

**Keywords:** ABCB1, anticancer agents, cancer stem cells, inhibitor of apoptosis proteins, leukaemia, TKIs, ABC-transporters, ATP-binding-cassette transporter, CML, chronic myeloid leukaemia, IAP, inhibitor of apoptosis protein, LMW-PTP, low molecular weight protein tyrosine phosphatases, MDR, multidrug resistance, OCT-1, organic cation transporter 1, Ph, Philadelphia chromosome, Shh, Sonic hedgehog, TG, thapsigargin, TKI, tyrosine kinase inhibitors, XIAP, X-linked inhibitor of apoptosis protein

## Abstract

The hallmark of CML (chronic myeloid leukaemia) is the *BCR* (breakpoint cluster region)*–ABL* fusion gene. CML evolves through three phases, based on both clinical and pathological features: a chronic phase, an accelerated phase and blast crisis. TKI (tyrosine kinase inhibitors) are the treatment modality for patients with chronic phase CML. The therapeutic potential of the TKI imatinib is affected by BCR–ABL dependent an independent mechanisms. Development of MDR (multidrug resistance) contributes to the overall clinical resistance. MDR involves overexpression of ABC -transporters (ATP-binding-cassette transporter) among other features. MDR studies include the analysis of cancer cell lines selected for resistance. CML blast crisis is accompanied by increased resistance to apoptosis. This work reviews the role played by the influx transporter OCT1 (organic cation transporter 1), by efflux ABC transporters, molecules involved in the modulation of apoptosis (p53, Bcl-2 family, CD95, IAPs (inhibitors of apoptosis protein)], Hh and Wnt/β-catenin pathways, cytoskeleton abnormalities and other features described in leukaemic cells of clinical samples and CML cell lines. An MDR cell line, Lucena-1, generated from K562 by stepwise exposure to vincristine, was used as our model and some potential anticancer drugs effective against the MDR cell line and patients’ samples are presented.

## INTRODUCTION

CML (chronic myeloid leukaemia) has an incidence 1–1.5 per 100000 inhabitants. It represents approximately 15% of all leukaemias diagnosed in adults with an onset at 40–60 years of age [1]. It is a myeloproliferative disease, and it is characterized by high levels of white blood cell counts, splenomegaly, weight loss, lethargy and anaemia. CML has an evolutive course comprising three clinical phases based on both clinical and pathological features [2]. The chronic phase is characterized by an increase in immature and mature myeloid elements, and retention of haematopoietic differentiation. The disease may then progress through an accelerated phase, or directly to an acute or blast phase when there are ≥30% blasts in the bone marrow or extramedullary blastic disease, and presents a very poor prognosis. During progression the main changes are seen in proliferation, activation of pathways that block myeloid differentiation, inhibition of tumour suppressor genes and enhancement of survival pathways [3].

The hallmark of CML is the *BCR—ABL* fusion gene, resulting from a chromosomal abnormality called Ph (Philadelphia chromosome) [4] and implicated in the pathogenesis of the disease. This chromosomal abnormality results from a reciprocal translocation between the chromosome 9 and chromosome 22 [t(9;22)(q34;q11)]. A chimaeric protein with 210-kDa, BCR–ABL, is typically found in patients with CML and is a constitutively active tyrosine kinase [5]. BCR–ABL then phosphorylates target proteins leading to the expansion of haematopoietic stem and progenitor cells through the activation of multiple signal transduction pathways.

The constitutively active BCR–ABL in CML cells provided an explanation for the initiation of the chronic phase and affords the possibility of using a target-orientated therapy. Treatment with imatinib mesylate, a TKI (tyrosine kinase inhibitor), has been shown to produce a pronounced and lasting response as a single agent in chronic phase CML patients. However, CML progression affects the outcome of imatinib therapy. The complete cytogenetic response rate for early chronic-phase patients placed on imatinib was found to be over 80%; for accelerated phase this was about 40% and during blast crisis the value falls to approximately 20%. This profile might result from the fact that the longer BCR–ABL is active before the initiation of therapy, the longer the cell is exposed to genomic instability [6].

Despite the fact that imatinib is a highly promising agent for treating CML, its therapeutic potential is limited due to amplification of the *BCR–ABL* gene or emergence of point mutations in BCR–ABL [7]. Although mutations outside the Abl kinase domain have been observed, the best studied mechanism is related to mutations in this domain where they may be located in different regions such as at the imatinib-binding site, at the ATP-binding site, in the activation loop, etc. [7,8]. Currently, approximately 100 different BCR–ABL kinase domain mutations have been described in imatinib resistant CML patients [9].

To overcome the resistance observed with imatinib treatment, other selective BCR–ABL TKIs have been developed [10,11]. Despite the development of second generation of TKIs, a minority of CML patients in chronic phase and a substantial proportion of patients in advanced phase are either initially refractory to TKIs or eventually develop resistance [9].

### Resistance mechanisms

**Figure 1:**
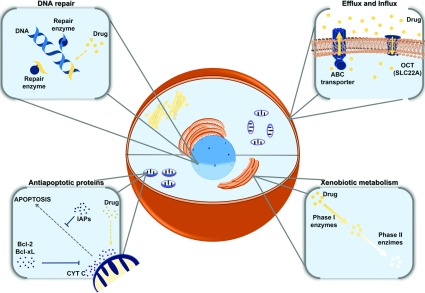
Mechanisms involved in the MDR phenotype

Although point mutations of BCR–ABL are frequently involved in TKIs resistance mechanisms, many other factors that abrogate an effective treatment with TKIs have been identified. Therefore TKIs resistance is a process involving BCR–ABL dependent and independent resistance mechanisms. BCR–ABL-independent mechanisms include non-adherence or intolerance to TKIs, decrease of intracellular TKIs influx, and the development of the phenomenon known as MDR (multidrug resistance). This phenomenon is a frequent cause of chemotherapy failure in cancer patients and it is characterized by cross-resistance to a broad range of anticancer drugs that may have different structures and mechanisms of action [12]. The better studied MDR mechanism involves the expression and activity of ABC transporters (ATP-binding-cassette transporter), but the resistance process is multifactorial and may involve mechanisms of repair, drug detoxification and resistance to apoptotic mechanisms (Figure 1).

### Using cell lines to understand resistance mechanisms

It is arguable whether cell lines are realistic representative of tumour cells [13]; however, they are still the best model to study a number of aspects of tumour biology and their use has contributed to the understanding of various mechanisms of resistance including the MDR phenotype.

The prototype of a human CML cell line is K562, derived from a pleural effusion of a patient suffering from CML in terminal blast crisis [14]. K562 was the first CML cell line with a persistent positive Ph chromosome after continuous long culture. K562 cells have been characterized as highly undifferentiated cells; these blasts are multipotential and haematopoietic malignant cells and have nearly 1.5 times the normal number of chromosomes. Studies on the surface membrane properties led to the conclusion that the K562 was a human erythroleukaemia line [15]. Despite the fact that K562 is by far the better studied model of human CML cell lines, other Ph positive cell lines obtained from CML patients in blast crisis have been described such as LAMA-84, KCL-22 and KYO-1 [16–18].

Studies using cell lines indicated that BCR–ABL activity contributes to a number of characteristics observed in CML such as apoptosis inhibition, disorganization of the cytoskeleton, decreased cellular adhesion, decreased cellular differentiation, etc. However, it must be taken into account that K562 represents a CML cell in blast crisis. Hopefully, with the advent of TKIs less CML patients will reach this phase.

One of the first studies on the establishment of a resistant tumour cell line was performed in 1983, with the resistance induction *in vitro* of K562 cells selected using increasing doses of the vinca alkaloid derivate, vincristine [19]. Since then a number of cell lines with the MDR phenotype have been generated and compared with leukaemic cells from CML patients, including cell lines resistant to imatinib [7].

### Modulation of influx transport in patient samples

The failure of TKIs to effectively inhibit leukaemic cells could result from impairment of drug uptake, so that it never reaches the inhibitory intracellular concentration. This could be because of a decrease in influx transport. Indeed, it has been reported that OCT1 (organic cation transporter 1), a member of the solute carrier transporters encoded by the *SLC22* gene family, mediates the transport of imatinib into cells [20]. Therefore the role of the organic cation transporter OCT1 has been studied in the context of imatinib uptake by leukemic cells obtained from CML patients [21,22]. It was found that intracellular imatinib uptake correlates with OCT1 expression or activity and may thus determine the therapeutic outcome [22]. However, neither nilotinib nor dasatinib cellular uptake was significantly affected by OCT-1 activity [20,23]. Studying samples obtained from 14 CML patients (five responsive and nine resistant to imatinib) it was observed that cells from all patients expressed higher *SLC22* mRNA levels compared with normal controls, furthermore, samples from resistant patients expressed even higher levels [24]. Data from our group, studying cell samples obtained from 57 CML patients showed that 79% of the samples presented reduced levels of mRNA for *SLC22A1*. Unfortunately, at that time no correlation was made with imatinib clinical responses (R.C. Maia, unpublished work). The importance of OCT1 was also studied in relation to a K562 cell line made resistant to imatinib, no difference being found in the expression and function of this transporter between the resistant and the K562 parental cell line [25]. However, using an MDR cell line, derived from K562 and selected with vincristine, an increase in the expression of OCT1 was found despite its resistance to imatinib [24].

### Modulation of efflux transport

Another way of keeping a drug from reaching an effective intracellular accumulation occurs through efflux mechanisms that diminish their retention inside the cells. ABC transporter family Pgp/ABCB1, MRP1 (multidrug resistance-associated protein 1)/ABCC1 and BCRP/ABCG2 are drug efflux transmembrane proteins that restrict intracellular accumulation of some drugs owing to their capacity to extrude substances from the cells [26]. Among these proteins, Pgp/ABCB1 was the first efflux pump transporter to be discovered [27]. Association of Pgp/ABCB1 with clinical drug resistance in CML was first described in 1990 [28,29]. The overexpression of some members of the ABC transporter family, leading to MDR phenotype, has been associated with the lack of sensitivity towards a number of unrelated drugs. Furthermore, the involvement of Pgp/ABCB1 and BCRP/ABCG2 overexpression and TKI efflux-mediated resistance has been reported [30].

Even before the clinical use of TKIs, various studies explored the expression/activity of efflux transporter proteins and their potential role in drug resistance in CML. Elevated Pgp/ABCB1 levels, analysed by Western blot and quantitative solid-phase plate radioimmunoassay, were observed in 55% of 198 samples obtained from CML patients. However, no correlation was found with disease progression or response to therapy [31]. Our group described the expression and activity of Pgp/ABCB1, and other efflux pumps, in cells obtained from CML patients at various phases of the disease [32]. In the blast phase, all samples exhibited Pgp/ABCB1 positivity in contrast to other CML phases. This finding could not be associated with the expression of the efflux transporter MRP1/ABCC1 since the proportion of MRP1/ABCC1 positive samples in the blast phase was lower [33]. These results are shown in Table 1.

**Table 1:** MDR profile of CML patients samples at different phases of the disease N, No. of samples; n, No. of positive samples; MDR, multidrug resistance; Rho, Rhodamine-123 efflux pump activity; CP, chronic phase; AP, accelerated phase; BP, blastic phase; Advanced, accelerated and blastic phases. The use of>1.1 cut off of the mean fluorescence intensity ratio threshold for rhodamine-123 efflux, ABCB1/Pgp and ABCC1/MRP1 expression was based on Lucena-1 (ABCB1 positive) and K562 (ABCB1 negative) cell lines measurement. p53 expression analysis was based on human tumour cell lines and peripheral blood mononuclear cells (PBMC) from healthy individuals. The cut-off value was established based on the ratio of mean fluorescence intensity levels of p53 stained in human tumour cell lines and PBMC. *XIAP and survivin levels were expressed through XIAP or Survivn/β-actin densitometric ratio normalized in relation to control using Western blot assay.

CML phase N.	Rho no.	ABCB1 no.	ABCC1 no.	p53 no.	Survivin* median	XIAP* median	Reference
CP=41	28	33	17				[33]
AP=07	04	06	04				
BP=08	04	08	01				
CP=12	09	09	08	03			[107]
AP=03	03	02	02	00			
BP=05	04	05	05	02			
CP=54				05			[57]
AP=07				04			
BP=11				08			
CP=13	08	12	11	06			[108]
Early CP=102	62	85/95	45/73				[32]
Late CP=70	52						
Advanced=73	46	56/64	21/34				
Early CP=30	25/26	22/30			1.23		[37]
Late CP=20	17/17	14/20			1.57		
Early CP=32	11/26					1.17	[83]
Late CP=07	02/06					1.05	
Advanced=09	04/06					1.24	

The resistant phenotype mediated by these transporters in CML can result from progression of the leukaemic process or can be induced by exposure to chemotherapeutic drugs including imatinib [34]. Patients treated with imatinib during 6–12 months had increased numbers of Pgp/ABCB1-positive peripheral blood cells, which correlated with Pgp/ABCB1 activity. Patients undergoing imatinib therapy for more than 6 months expressed, in addition, other efflux transporter proteins such as MRP1/ABCC1 and BCRP/ABCG2 [35].

A number of studies involving ABC transporters in CML have been performed using cell lines. There are two main strategies for obtaining resistant cell lines in cells originally susceptible to chemotherapy: by transfection of specific ABC transporter genes [36], or as a result of selection of resistant cells following exposure to increasing concentrations of cytotoxic drugs. While transfection of the ABC transporter gene is a rapid method, which is widely used for expression of the MDR phenotype, with this approach the cell only expresses the MDR-specific protein, differently to the situation in which the cell lines are selected *in vitro* or in patients receiving progressive doses of chemotherapeutic drugs. In such cases, other mechanisms of resistance can be selected in parallel and as a result it is also possible to observe the expression of more than one ABC transporter. As mentioned earlier multiple mechanisms may confer an MDR phenotype. Similar to what is observed during patients’ treatment [32,37], cell lines selected *in vitro* may display multiple resistance phenotypes that vary during different stages of the selection process [38].

The same selection technique, using stepwise increases of a given chemotherapeutic drug, has been employed by a number of workers to obtain new resistant cell lines. Our group compared two resistant cell sublines derived from K562 and generated by stepwise selection in vincristine or daunorubicin [39]. The resistant lines were designated Lucena-1 and FEPS, respectively, and depict various resistance strategies that can be generated in MDR cell lines using the same parental cell but different inducing agents. These results are in accordance to what has been previously observed by other authors using vincristine and doxorubicin-resistant cell lines derived from the same parental lymphoid leukaemia cell line [40].

The K562 derived sublines, Lucena-1 and FEPS, were resistant to vincristine, daunorubicin and imatinib and sensitive to cisplatinum, conferring to these cells the MDR phenotype [39,41]. The lack of susceptibility has been attributed to the overexpression of Pgp/ABCB1 in both cell lines and additionally, FEPS overexpression of MRP1/ABCC1 as well. But a number of other characteristics have been reported in the resistant subline Lucena-1 (Table 2).

**Table 2:** Mechanisms related to MDR in Lucena-1 cell line High, high levels when compared with K562 cell line; Low, low levels when compared with K562 cell line; Equal, no statistical significance when compared with K562 cell line; Oct-4(POU5F1), POU class 5 homeobox 1; RT-qPCR, Real-time quantitative PCR analysis; CDNB, 1-Chloro-2,4-dinitrobenzene; Gli1, GLI family zinc finger 1; Shh, Sonic hedgehog, Ptch-1, patched 1; SUFU, Suppressor of fused homologue; LMW-PTP, Low molecular weight protein tyrosine phosphatase. A proteomic profile of Lucena-1 cell line can be observed elsewhere [24].

Mechanism	Lucena-1	Method	Reference
*ABCB1*(*MDR-*1) mRNA	High	RT-PCR	[41]
ABCB1(Pgp)	High	Immunofluorescence	[41]
ABCC1 (MRP1)	Equal (negative)	Immunofluorescence	[41]
*ABCC1*(*MRP1*) mRNA	High (1,5×)	RT-qPCR	[99]
*ABCG2*(*BCRP*) mRNA	High (4×)	RT-qPCR	[99]
*ABCG2*(*BCRP*) mRNA	Equal	RT-qPCR	[24]
Alpha-tubulin	High	Western blot	[105]
*BCR-ABL* mRNA	High	RT-qPCR	[24]
Catalase activity	High	H_2_O_2_ substrate	[105,109]
*Cox-2* mRNA	High	RT-qPCR	[110]
Ecto-5 -nucleotidase activity	High	AMP substrate	[91]
Gli1	High	Western blot	[97]
*Gli1* mRNA	High (7×)	RT-qPCR	[97]
IL-8 production	Low	Flow cytometry	[39]
LMW-PTP	High	Western blot	[106]
LMW-PTP activity	High (7×)	Phosphotyrosine peptide substrate	[106]
*OCT1* (*SLC22A1*) mRNA	High	RT-qPCR	[24]
*Oct-4*(*POU5F1*) mRNA	High (3×)	RT-qPCR	[99]
p53	Equal	Flow cytometry	[39]
Ptch-1	High	Western blot	[97]
Shh	High	Western blot	[97]
*SUFU* mRNA	Low (2×)	RT-qPCR	[97]
*TP53* mRNA	High	RT-qPCR	[110]
β-catenin	High	Flow cytometry	[98]
*β-catenin* mRNA	High	RT-qPCR	[98]

MDR cell lines, generated by different groups were important to demonstrate that imatinib is a substrate transported by Pgp/ABCB1 [35,42,43]. Work by our group, using the MDR cell lines, Lucena-1 and FEPS, silenced for *ABCB1* suggested that additional mechanisms may also play a role in imatinib resistance [39]. Other authors have shown, using CML cell lines, that BCRP/ABCG2 is capable of transporting TKIs [44]. These results are in accordance to observations made using leukaemic cells from CML patients.

### Overcoming MDR mediated by ABC transporters

To overcome resistance, a number of substances capable of inhibiting the activity of ABC transporters known as chemosensitizers or reversers have been assayed since the first description by Tsuruo et al. using verapamil [45]. Cyclosporin A was also found to be an efficient chemosensitizer *in vitro* [46]. Both Verapamil and cyclosporin A are ABCB1 substrates and it is believed that they inhibit by competition [47]. It has been reported that TKIs, at high concentrations, may inhibit Pgp/ABCB1 activity also playing the role of chemosensitizers [48].

However, the clinical use of verapamil and other reversers such as cyclosporine A, produced disappointing results [49–51]. Clinical trials are now underway with new generation inhibitors [47,52]. Independently of its use *in vivo*, reversers may be used *in vitro*, to ascertain whether patients’ samples display an active ABC transporter mechanism.

### Modulation of survival mechanisms

Independently of resistance induction as a result of exposure to chemotherapeutic drugs, the transition towards CML blast crisis is accompanied by increased resistance to apoptosis. A number of aspects of the apoptotic process have been analysed in leukaemic cells obtained from patients and cell lines. Among them, the role played by p53 as it is well known that the activation of this gene promotes apoptosis and, conversely, disruption of this pathway can lead to tumour development. Furthermore, loss or mutations of this suppressor gene may affect the outcome observed when leukaemic cells are exposed to a variety of stimuli and chemotherapeutic drugs. Other components of the apoptotic process such as members of the Bcl-2 family, involved in the regulation of the intrinsic pathway, and members of death receptor pathway such as CD95 were also studied in CML. Finally, downstream of the apoptotic pathway, the IAPs (inhibitors of apoptosis proteins) are capable of regulating caspases and were found to be increased during the course of CML.

### p53

During CML progression, loss of p53 contributes to blast transformation of p210 BCR–ABL-expressing haematopoietic cells [53]. Several studies have reported structural alterations of chromosome 17, mutations of *p53* gene and p53 protein expression in blast phase [54–56]. Our group detected *p53* expression in samples from CML patients analysed by flow cytometry using anti-p53 monoclonal antibodies and an increased expression could be observed as the disease progressed to the blast phase (Table 1). A positive relationship was found between *p53* expression and high risk determined by Sokal score system, which is calculated using peripheral blood blast number, platelet count, spleen size and age in low-intermediate- or high-risk groups at the time of diagnosis [57].

Different human CML cell lines differ in relation to p53 [58]. When p53 was studied using the MDR cell lines Lucena-1 and FEPS compared with K562 no difference was observed.

### Bcl-2 family of anti-apoptotic proteins

Cell death, as a result of mitochondria-regulated apoptosis promoted by the release of cytochrome *c*, is dependent on members of the Bcl-2 family. This family is characterized by the pro-apoptotic proteins Bax and Bim and the antiapoptotic Bcl-2 and Bcl-X_L_.

Leukaemic cells from CML patients display antiapoptotic mechanisms that lead to cell survival [59]. It has been reported that samples from CML patients express elevated levels of Bcl-X_L_ and Mcl-1. Some of these effects are directly related to BCR–ABL as it activates STAT 5 increasing the expression of Bcl-2 and Bcl-X_L_ [60–63]. However, no increase in Bcl-2 levels in patients’ samples has been reported. Additionally, BCR–ABL inhibits caspase activation after the release of cytochrome *c* [64].

However, increased cell survival is in fact the outcome resulting from the balance between antiapoptotic, for example Bcl-X_L_, and proapoptotic molecules, such as BAX. In samples from CML patients, the levels of BAX are maintained but the ratio BAX/Bcl-X_L_ varies during disease progression. Moreover, the level of Bcl-X_L_ decreases when TKIs are used [65]. In addition, other molecules capable of counteracting the antiapoptotic effect, such as Bim, are expressed in low amounts in CML cells but their expression is increased when BCR–ABL is inhibited [66].

A similar situation, which is dependent on a balance between antiapoptotic and pro-apoptotic molecules, is also observed in cell lines. K562 expresses low levels of Bim, but higher levels of these molecules are obtained after the treatment with imatinib that regulates BCR–ABL [67]. In accordance with the observations of samples from CML patients, no increased levels of Bcl-2 were observed in K562 cells, and a similar result was observed in its MDR sublines Lucena-1 and FEPS [39,68].

### CD95 (FAS)

CD95 also known as APO-1 or Fas receptor is a surface molecule that acts as a death receptor and is capable of transducing apoptotic signals into the cell after binding to its physiological ligand CD95L (FasL/APO-1L). When the expression of CD95 was studied on cells obtained from CML patients, despite the variability, the proportion of CML cells positive for this receptor was higher than what was seen when normal bone marrow cells were analysed, and it has been reported that these levels increase after interferon-alpha therapy [69,70]. CD95 expression does not seem to correlate with susceptibility to apoptosis, particularly in CML patients in blast crisis that are resistant to apoptosis induction via this receptor [70]. However, independently of the stage of the disease, cells from CML patients seem to be more refractory to CD95 induced apoptosis compared with other haematological malignancies [71].

CD95 is down-regulated during cancer progression and it has been proposed that CD95 loss is partly responsible for tumour evasion of the immune system. However, it has been suggested that CD95 could actually promote the growth of tumours, stimulated by their own CD95L [72]. A possible explanation for this observation is that tumour growth and apoptosis induction involves different pathways and are responsive to different thresholds [73].

As mentioned earlier the cell line K562 represents CML cells in the blast phase. There are conflicting reports related to CD95 expression at the surface of K562 cells. Some authors could not detect its expression [74,75], whereas other found a low expression compared with other leukaemia cell lines and described that these cells were resistant to apoptosis induction [76]. When K562 cells were transfected and expressing CD95, they were still protected from CD95-mediated cell death [74,75] and it has been suggested that Abl kinase acts as a negative regulator of cell death. If this is the case, during MDR, the induction Abl activation could occur and would be responsible for the lack of apoptosis via CD95–CD95L [76].

In our hands, K562 and the MDR Lucena-1 expressed similar levels of CD95, whereas the other MDR counterpart, FEPS, expressed significantly lower levels. Furthermore, silencing *ABCB1* in these cell lines did not modify CD95 expression, suggesting that the two events are independent [39].

### IAPs

IAPs (inhibitor apoptosis proteins) are characterized by their ability to block apoptosis through the inhibition of mitochondrial-dependent and -independent apoptotic pathways. IAPs overexpression has been considered a poor prognostic marker in several types of cancer [77]. Besides suppression of apoptosis IAP genes are involved in a number of other cellular functions. This is the case of IAP survivin, which besides regulating cell division [78] has an important role in chemoresistance of malignancies including CML [79]. In cells from CML patients, high levels of survivin expression correlate with BCR–ABL expression levels suggesting that survivin in CML could be regulated by *BCR–ABL* [80]. Our group also detected higher levels of survivin expression in cells from CML patients in late chronic-phase CML compared with newly diagnosed (early chronic phase CML patients) (Table 1) [37]. Survivin expression was strongly and positively correlated with Pgp/ABCB1 expression, but not with Pgp/ABCB1 activity. These findings suggest that Pgp/ABCB1 may be associated with drug-resistance mechanisms independently of its role in drug efflux [81]. Our study suggests that the significant correlation between Pgp/ABCB1 and survivin in late chronic phase CML, but not in early chronic phase CML, indicates a possible role for this association in the evolution of CML [37].

Another IAP-denominated XIAP (X-linked inhibitor of apoptosis protein), in contrast to survivin, is widely expressed in normal tissues but, similar to survivin, XIAP overexpression in cancer is usually associated with an unfavourable prognosis [82]. However, little is known about XIAP expression in CML patients. Our group observed high levels of XIAP expression in 16 out of 32 (50%) samples from CML patients at early chronic phase and five out of nine samples (55.5%) at advanced phases (Table 1). A positive correlation between XIAP and Pgp/ABCB1 expression was also observed [83].

IAPs have also been studied in the context of CML cell lines. When the leukaemic cell line K562 and its MDR counterpart were studied in relation to leukaemia infiltration in a xenograph model, it was observed that resistant cells showed an increased invasive capability when compared with parental cells [84]. Furthermore, the same group reported that Pgp and cIAP were overexpressed in the MDR cell line and co-localized with PKC (protein kinase C)-ϵ [84].

Survivin and XIAP were investigated during the process of resistance induction in K562 cells by a high dose of vincristine. Despite a progressive increase in the expression of survivin the same did not occur with XIAP expression. However, a concomitant overexpression of Pgp/ABCB1 and survivin was observed, preventing cell death [85]. The association between Pgp/ABCB1 expression and survivin agrees with those from samples obtained from CML patients [81]. In addition, cytoplasmic co-localization of Pgp/ABCB1 and survivin was observed in K562 cells made resistant through exposure to a high dose of vincristine, thus suggesting a functional association between these two proteins [85].

During apoptosis induction by imatinib or ara-C in K562 cells, survivin translocates to the nucleus. This did not occur when the MDR cell line Lucena-1, that overexpresses Pgp/ABCB1, was studied. In this case, the low rate of apoptosis was related to survivin cytoplasmatic localization [86].

Survivin also confers resistance to apoptosis induced by other non-TKIs drugs such as idarubicin in K562 cell line. It is well known that, despite TKIs incorporation in the treatment of blast phase CML, other chemotherapeutic agents, such as the anthracycline idarubicin in combination or not with imatinib, are also used to treat CML in blast phase. These *in vitro* results using cell lines suggest that, imatinib and idarubicin in association, although synergistic, may not produce such a good therapeutic result because survivin expression contributes to imatinib and idarubicin resistance phenotype [87].

### Calcium homoeostasis in CML and the apoptotic process

Alterations in calcium intracellular localization and mobilization are important as an excess of cytosolic calcium may trigger apoptosis or necrosis. Leukaemic cells from CML patients show decreased calcium mobilization induced by ATP, ionomycin or InsP3 [88]. However, no difference between cells from normal controls and from CML patients was observed in relation to the production of ROS (reactive oxygen species) [88].

Cells overexpressing Pgp/ABCB1 show several differences related to calcium homoeostasis [89]. Comparing the cell lines K562 and its MDR counterpart, it was observed that Lucena-1, similar to other Pgp/ABCB1 overexpressing cells, is resistant to the calcium ATPase inhibitor TG (thapsigargin). Treatment with the classical MDR modulators cyclosporin A and verapamil could not reverse this resistance, suggesting that the absence of effect was not due to TG extrusion [68]. A number of studies suggested that TG resistance due to Pgp/ABCB1 overexpression is the result of a more complex process than extrusion of the drug [90]. These differences may add to the resistance observed in these cells.

In accordance with the lack of calcium mobilization induced by TG, it was observed that ATP did not increased intracellular calcium levels. ATP may be released in areas of cell destruction regulating tumour survival. Studying K562 and Lucena-1 cells, it was observed that ATP and its products from hydrolysis were able to induce apoptosis. Similarly, UTP induced apoptosis but this characteristic was not shared by its products of hydrolysis, UDP and UMP. This cytotoxic effect produced by ATP occurred independently of the participation of the receptor P2×7, which is related to plasma membrane permeabilization. Altogether, this suggests that UTP and ATP can promote apoptosis, independent of MDR phenotype, and this mechanism does not involve P2×7 [91].

### Cancer stem cells and MDR

Cancer stem cells have the capacity of self-renewal and asymmetric division, and they are believed to promote tumour recurrence and to originate metastasis. The normal stem cell is quiescent, needs to remain viable and intact during the entire life of the individual, and possess a number of protective mechanisms. The presence of cancer stem cells have been described in CML patients [92]. The tumour initiating cell, shares a number of normal stem cell features. They are resistant towards classical chemotherapy that relies on their action towards actively proliferating cells and induces cytotoxicity via apoptosis. Furthermore, haematopoietic stem cells express efflux transporters from the ABC family, mainly Pgp/ABCB1, which confers protection against xenobiotics including anti-cancer agents [93].

In CML, CD34^+^ haematopoietic precursors overexpress a number of ABC transporters. This expression seems to be driven by c-MYC, which is in accordance with the demonstration that BCR–ABL is able to increase c-MYC expression. The increased expression of ABC drug transporters by the BCR–ABL/c-MYC network increases their self-renewal potential and lower sensitivity to drug treatments [94].

Due to the stem cell characteristics of leukaemic stem cells, a different therapeutic approach would be to selectively inhibit pathways connected with self-renewal [95]. This is an important point when one considers that most anti-cancer drugs fails to eliminate leukaemia stem cells in CML particularly at the blast phase. Strictly regulated pathways such as Wnt, Hedgehog and Notch regulate self-renewal and survival of HSC (heat-shock cognate), and aberrant Hedgehog signalling has been described in the leukaemic stem cell population [96]. A cross-regulation of signalling network involving Shh (Sonic hedgehog), Wnt, Notch and Hox has been observed and blast-crisis has been associated with proactive Shh and Wnt signalling, down-regulated Hox and deregulated Notch pathways [96].

A correlation between Hedgehog pathway and MDR phenotype in CML cell lines has been described [97]. The transfection of K562 cells with the SmoM2-GFP plasmid, which expressed a constitutively Smo variant that stimulates the Hh pathway, led to increased resistance to vincristine. Indeed, when the MDR cell line Lucena-1 was transfected with the Gant61 plasmid, that represses the Hh pathway, these cells were rendered more susceptible to vincristine, mitoxantrone and doxorubicin [97]. The data suggest that Hh pathway is involved in the MDR phenotype.

Previous reports have associated the Wnt/β-catenin pathway with Pgp/ABCB1 regulation in cancer. The same pathway has been reported in patients in which CML is progressing to the acute phase. These observations led Correa et al. [98] to explore the regulation of Pgp/ABCB1 by Wnt/β-catenin pathway in CML cells. Besides the high expression of Pgp/ABCB1, it was reported that the MDR cell line Lucena-1 also expressed more β-catenin than K562. The ChIP (chromatin immunoprecipitation) assay for β-catenin displayed a high content of bound Pgp/ABCB1 in Lucena-1 cells. In addition, the silencing of β-catenin or Wnt1 using siRNA decreases the level of Pgp/ABCB1 in Lucena-1. The opposite happened when these cells were treated with LiCl, which induces the nuclear translocation of β-catenin. The data support the hypothesis that Wnt/β-catenin pathway has a crucial role in Pgp/ABCB1 expression in Lucena-1, an MDR cell line [98].

Other characteristics of cancer stem cells have been described in K562 and its MDR counterparts. It has been reported that both Lucena-1 and the parental K562 cells presented the profile CD34^+^CD38^−^, which is a hallmark of the early stages of haematopoietic stem cells [99], but there is some controversy related to K562 expression. The stem cell markers Sox, Nanog and Oct-4 were elevated in a another MDR cell line selected using doxorubicin when compared with the parental K562 [100]. Oct-4/POU5F1 is a member of the POU family of transcription factors and its expression had been associated with cancer stem cells. Oct-4 was also found to be three times more expressed in Lucena-1 (originally generated in presence of vincristine) than in K562 [99]. Besides, there is an Oct-4 core sequence (ATGCAAAT) in the promoters of Pgp/ABCB1, ABCC1 and ABCG2. The mRNA of these ABC transporters were studied in Lucena-1 [99]. In cancer stem cells, Oct-4 appears to be involved in the increased expression of ABC transporters [101]. In this context, it is reasonable to assume that Oct-4 has a similar role in MDR cell lines. A different group, using an adenocarcinoma model, presented data suggesting that the Oct-4 protein is able to activate β-catenin. Furthermore, β-catenin is able to translocate into the nucleus and activate its target genes [102]. Collectively, these results support the possibility of β-catenin being also regulated by Oct-4.

### Using proteomics to identify the MDR phenotype

To better understand the mechanisms responsible for the MDR phenotype, the differential proteome of K562 and its resistant subline Lucena-1 was analysed through mass spectrometry. Among the 36 differentially translated proteins identified, 14 proteins were down-expressed and 22 proteins were over-expressed in Lucena-1 cells. It should be stressed that the number of differentially translated proteins is probably larger than what was found. However, the ones described form a picture of different pathways involved in MDR. According to the *in silico* analysis, the proteins related to Cellular Function and Maintenance; Interaction and Small Molecule Biochemistry; DNA Replication, Recombination, and Repair; Cell-to-Cell Signalling and Cell Death were among the most relevant in the context of resistance. Besides the high expression of Pgp/ABCB1, two other highly expressed proteins, MCM7 and LRPPRC, identified in Lucena-1 were validated in CML patients resistant to imatinib [24].

### Other characteristics of CML cells

Actin and tubulin are major structural proteins of the cytoskeleton involved in cell division and cell movement. A number of alterations in the cytoskeleton of CML cells have been reported, some of which may affect the functions of these cells such as cellular movement, binding of chemo-attractants and cell cycle control. Furthermore, it has been reported that p210 BCR–ABL binds to actin and phosphorylates cytoskeletal proteins [103]. However, inhibiting the kinase activity of these cells with imatinib inhibited proliferation but not defects on adhesion nor migration which seems to be a direct effect of the interaction between BCR–ABL and actin. In addition to that, the development of MDR with the overexpression of Pgp/ABCB1 leads to alterations of various cytoskeleton elements. These results suggested, at least when transformed embryo fibroblasts were used, that these changes were essential for evolution of MDR mechanisms [104]. Using CML cell lines, it was observed that Lucena-1 had increased amounts of alpha-tubulin when compared with K562 cells [105]; however it was difficult to dissociate this effect from the fact that Lucena-1 cells had been selected using vincristine.

LMW-PTP (low molecular weight protein tyrosine phosphatases) have been related to a poor prognosis in some cancers. The LMW-PTP are a family of proteins associated with cytoskeleton rearrangement, cell growth and immune response. A relation between the expression of these proteins to MDR phenotype was studied using K562 and Lucena-1 as a model. It was observed that Lucena-1 expressed more LMW-PTP than K562. In addition, the transfection of LMW-PTP into K562 leads to vincristine resistance. Conversely, the silencing of LMW-PTP in Lucena-1 reverses the MDR phenotype. This was followed by capase-3 activation. Probably, the mechanism of LMW-PTP responsible to this resistance is related to Src and BCR–ABL activation, since the silencing of LMW-PTP decreases the phosphorylation of these proteins [106].

**Figure 2:**
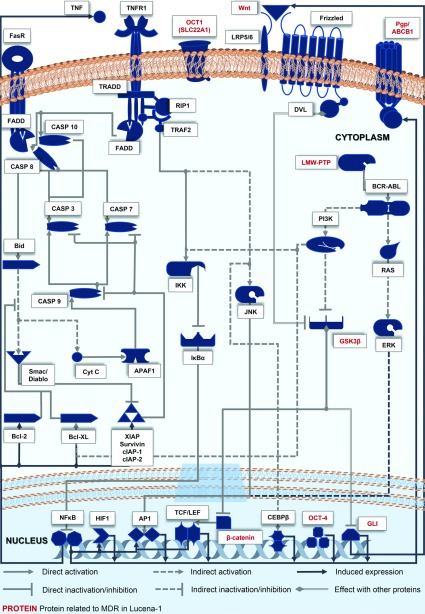
Signalling pathways involved in MDR phenotype: the case of Lucena-1 cell line A model of crosstalk among the pathways and their related proteins that lead to the MDR phenotype in Lucena-1 cells. Based on the canonical pathways at KEGG database [119] for the transcription factors of the *ABCB1* gene described by Scotto [120] and the reports cited within this review. FasR, Fas [TNF (tumour necrosis factor) receptor superfamily, member 6]; TNFR, tumour necrosis factor receptor superfamily, member 1; FADD, Fas (TNFRSF6)-associated via death domain; TRADD; TNFRSF1A-associated via death domain; RIP1, Receptor (TNFRSF)-interacting serine–threonine kinase 1; TRAF2, TNF receptor-associated factor 2; CASP 10, caspase 10, apoptosis-related cysteine peptidase; CASP 8, caspase 8, apoptosis-related cysteine peptidase; CASP 3, caspase 3, apoptosis-related cysteine peptidase; CASP 7, caspase 7, apoptosis-related cysteine peptidase; CASP 9, caspase 9, apoptosis-related cysteine peptidase; Bid, BH3 interacting domain death agonist; BCL-2, B-cell CLL/lymphoma 2; Bcl-X_L_, BCL2-like 1; Smac/Diablo, diablo, IAP-binding mitochondrial protein; XIAP, X-linked inhibitor of apoptosis; Survivin, baculoviral IAP repeat containing 5; cIAP-1, baculoviral IAP repeat containing 2; cIAP-2, baculoviral IAP repeat containing 3; NF-κB, nuclear factor of kappa light polypeptide gene enhancer in B-cells; HIF1, hypoxia inducible factor 1, alpha subunit; AP1, Transcription factors composed of members of the Jun and Fos families; TCEF/LEF, transcription factors of the T-cell factor/lymphoid enhancer factor family; B-catenin, catenin (cadherin-associated protein), beta 1; CYT C, cytochrome *c*, somatic; APAF1, apoptotic peptidase activating factor 1; IκB, nuclear factor of kappa light polypeptide gene enhancer in B-cells inhibitor, alpha; IKK, kinases of inhibitor of kappa light polypeptide gene enhancer in B-cells; Wnt, wingless-type MMTV integration site family; LRP5/6, low-density lipoprotein receptor-related protein 5 and protein 6; Frizzled, receptors of frizzled family; DVL, dishevelled, dsh homologue 1 (*Drosophila*); PI3K, phosphatidylinositol-4,5-bisphosphate 3-kinase; BCR–ABL, fusion protein encoded by BCR and c-abl oncogene 1, non-receptor tyrosine kinase (ABL) genes; JNK, mitogen-activated protein kinase 8; GSK3B, glycogen synthase kinase 3 beta; LMW-PTP, low molecular weight protein tyrosine phosphatases; RAS, Ras superfamily of proteins; ERK (extracellular-signal-regulated kinase), mitogen-activated protein kinase 1; GLI,GLI family zinc finger; OCT-4,POU class 5 homeobox 1; CEPBeta CCAAT/enhancer binding protein (C/EBP), beta.

A summary of signalling pathways that have been described in the MDR cell line Lucena-1 is shown in Figure 2, and some of the characteristics present in Lucena-1 compared to the parental cell K562 is shown Table 2.

### Using cell lines for the identification of new anticancer agents

Cells lines and their MDR counterparts have been used as *in vitro* models to identify novel anticancer drugs capable of overcoming the MDR phenotype. At the same time this kind of study helps to disclose some characteristics that may affect the susceptibility of MDR cells. Many different groups have been studying the effect of various substances with activity against CML cell lines and patients leukemic cells. Table 3 describes some of the substances tested and shown to have activity against MDR CML cells.

**Table 3:** Screening for cytotoxic or cytostatic drugs effective against CML cells exhibiting the MDR profile PBMC, peripheral blood mononuclear cell; CPT-11, 7-ethyl-10-[4-(1-poperidino)-1-piperidino] carbonoxyl camptothecin (irinotecan); PI, propidium iodide.

Drug	Model	Method	Reference
Betulinic acid	K562 and Lucena-1	^3^H thymidine incorporation	[111]
Pomolic acid	Patient's sample, K562 and Lucena-1 and PBMC	^3^H thymidine incorporation and Annexin-V/PI staining	[107,111]
Oleanolic acid	K562 and Lucena-1	^3^H thymidine incorporation	[111]
Euscaphic acid	K562, Lucena-1 and other cell lines	MTT assay, PI staining and evaluation of caspase-3	[112]
Tormentic acid	K562 and Lucena-1	MTT assay	[112]
2α-acetyl tormentic acid	K562 and Lucena-1	MTT assay	[112]
3-acetyl tormentic acid	K562 and Lucena-1	MTT assay	[112]
Sodium orthovanadate	K562, Lucena-1 and other cell lines	Exclusion by trypan blue	[113]
Methylene Blue	K562, Lucena-1 and PBMC	^3^H thymidine incorporation and MTT assay	[114]
CPT11	Patient's sample, K562 and Lucena-1	Annexin-V/PI staining and MTT assay	[115]
Microcystin	K562 and Lucena-1	Exclusion by trypan blue and MTT assay	[105]
Acetylsalicylic acid	K562, Lucena-1 and PBMC	Annexin-V/PI staining and exclusion by trypan blue	[110]
Pterocarpans	K562, Lucena-1 and PBMC	MTT assay	[116,117]
*Ortho*-quinone	K562, Lucena-1 and PBMC	MTT assay	[116]
Pentacyclic 1,4-naphthoquinones of type 1	Patient's sample, K562, Lucena-1, other cell lines and PBMC	Annexin-V staining and MTT assay	[117]
LQB-118	Patient's sample, K562, Lucena-1, other cell lines and PBMC	Annexin-V/PI staining, MTT assay and evaluation of caspase-3	[108,118]

Anticancer drugs, as the ones reported here, indicate that is possible to overcome the MDR phenotype *in vitro.* More translational work is necessary to transport this information to the clinical practice.

### Conclusion

Despite not representing exactly what is observed with patients’ samples and being removed from the influences of their microenviroment, the use of CML cell lines can provide useful information regarding the biology of these cells and candidate substances to overcome the MDR phenotype in CML.

## References

[1] Jabbour E., Kantarjian H. (2012). Chronic myeloid leukemia: 2012 update on diagnosis, monitoring, and management. American J. Hematol..

[2] Faderl S., Kantarjian H. M., Talpaz M. (1999). Chronic myelogenous leukemia: update on biology and treatment. Oncology (Williston Park, NY).

[3] Perrotti D., Jamieson C., Goldman J., Skorski T. (2010). Chronic myeloid leukemia: mechanisms of blastic transformation. J. Clin. Invest..

[4] Nowell P. C., Hungerford D. A. (1960). Chromosome studies on normal and leukemic human leukocytes. J. Natl. Cancer Inst..

[5] Kurzrock R., Gutterman J. U., Talpaz M. (1988). The molecular genetics of Philadelphia chromosome-positive leukemias. New Engl. J. Med..

[6] Radich J. P. (2007). The Biology of CML blast crisis. Hematology/the Education Program of the American Society of Hematology. American Society of Hematology. Education Program.

[7] Mahon F. X., Deininger M. W., Schultheis B., Chabrol J., Reiffers J., Goldman J. M., Melo J. V. (2000). Selection and characterization of BCR-ABL positive cell lines with differential sensitivity to the tyrosine kinase inhibitor STI571: diverse mechanisms of resistance. Blood.

[8] Vaidya S., Ghosh K., Vundinti B. R. (2011). Recent developments in drug resistance mechanism in chronic myeloid leukemia: a review. Eur. J. Haematol..

[9] Ernst T., Hochhaus A. (2012). Chronic myeloid leukemia: clinical impact of BCR-ABL1 mutations and other lesions associated with disease progression. Semin. Oncol..

[10] Kantarjian H. M., Giles F., Gattermann N., Bhalla K., Alimena G., Palandri F., Ossenkoppele G. J., Nicolini F.-E., O’Brien S. G., Litzow M. (2007). Nilotinib (formerly AMN107), a highly selective BCR-ABL tyrosine kinase inhibitor, is effective in patients with Philadelphia chromosome-positive chronic myelogenous leukemia in chronic phase following imatinib resistance and intolerance. Blood.

[11] Talpaz M., Shah N. P., Kantarjian H., Donato N., Nicoll J., Paquette R., Cortes J., O’Brien S., Nicaise C., Bleickardt E. (2006). Dasatinib in imatinib-resistant Philadelphia chromosome-positive leukemias. New Engl. J. Med..

[12] Gottesman M. M. (2002). Mechanisms of cancer drug resistance. Annu. Rev. Med..

[13] Ertel A., Verghese A., Byers S. W., Ochs M., Tozeren A. (2006). Pathway-specific differences between tumor cell lines and normal and tumor tissue cells. Mol. Cancer.

[14] Lozzio C. B., Lozzio B. B. (1975). Human chronic myelogenous leukemia cell-line with positive Philadelphia chromosome. Blood.

[15] Andersson L. C., Nilsson K., Gahmberg C. G. (1979). K562–a human erythroleukemic cell line. International journal of cancer. J. Int. Cancer.

[16] Seigneurin D., Champelovier P., Mouchiroud G., Berthier R., Leroux D., Prenant M., McGregor J., Starck J., Morle F., Micouin C. (1987). Human chronic myeloid leukemic cell line with positive Philadelphia chromosome exhibits megakaryocytic and erythroid characteristics. Exp. Hematol..

[17] Kubonishi I., Miyoshi I. (1983). Establishment of a Ph1 chromosome-positive cell line from chronic myelogenous leukemia in blast crisis. Int. J. Cell Cloning.

[18] Ohkubo T., Kamamoto T., Kita K., Hiraoka A., Yoshida Y., Uchino H. (1985). A novel Ph1 chromosome positive cell line established from a patient with chronic myelogenous leukemia in blastic crisis. Leuk. Res..

[19] Tsuruo T., Iida H., Ohkochi E., Tsukagoshi S., Sakurai Y. (1983). Establishment and properties of vincristine-resistant human myelogenous leukemia K562. Gann.

[20] White D. L., Saunders V. A., Dang P., Engler J., Zannettino A. C. W., Cambareri A. C., Quinn S. R., Manley P. W., Hughes T. P. (2006). OCT-1-mediated influx is a key determinant of the intracellular uptake of imatinib but not nilotinib (AMN107): reduced OCT-1 activity is the cause of low *in vitro* sensitivity to imatinib. Blood.

[21] Nestal de Moraes G., Souza P. S., Costas F. C. de F., Vasconcelos F. C., Reis F. R. S., Maia R. C. (2012). The interface between BCR–ABL-dependent and -independent resistance signaling pathways in chronic myeloid leukemia. Leuk. Res. Treat..

[22] Wang L., Giannoudis A., Lane S., Williamson P., Pirmohamed M., Clark R. E. (2008). Expression of the uptake drug transporter hOCT1 is an important clinical determinant of the response to imatinib in chronic myeloid leukemia. Clin. Pharmacol. Therap..

[23] Hiwase D. K., Saunders V., Hewett D., Frede A., Zrim S., Dang P., Eadie L., To L. B., Melo J., Kumar S. (2008). Dasatinib cellular uptake and efflux in chronic myeloid leukemia cells: therapeutic implications. Clin. Cancer Res..

[24] Corrêa S., Pizzatti L., Du Rocher B., Mencalha A., Pinto D., Abdelhay E. (2012). A comparative proteomic study identified LRPPRC and MCM7 as putative actors in imatinib mesylate cross-resistance in Lucena cell line. Proteome Sci..

[25] Hirayama C., Watanabe H., Nakashima R., Nanbu T., Hamada A., Kuniyasu A., Nakayama H., Kawaguchi T., Saito H. (2008). Constitutive overexpression of P-glycoprotein, rather than breast cancer resistance protein or organic cation transporter 1, contributes to acquisition of imatinib-resistance in K562 cells. Pharm. Res..

[26] Chen K. G., Sikic B. I. (2012). Molecular pathways: regulation and therapeutic implications of multidrug resistance. Clin. Cancer Res..

[27] Juliano R. L., Ling V. (1976). A surface glycoprotein modulating drug permeability in Chinese hamster ovary cell mutants. Biochim. Biophys. Acta.

[28] Kuwazuru Y., Yoshimura A., Hanada S., Ichikawa M., Saito T., Uozumi K., Utsunomiya A., Arima T., Akiyama S. (1990). Expression of the multidrug transporter, P-glycoprotein, in chronic myelogenous leukaemia cells in blast crisis. Br. J. Haematol..

[29] Herweijer H., Sonneveld P., Baas F., Nooter K. (1990). Expression of mdr1 and mdr3 multidrug-resistance genes in human acute and chronic leukemias and association with stimulation of drug accumulation by cyclosporine. J. Natl. Cancer Inst..

[30] Dohse M., Scharenberg C., Shukla S., Robey R. W., Volkmann T., Deeken J. F., Brendel C., Ambudkar S. V, Neubauer A., Bates S. E. (2010). Comparison of ATP-binding cassette transporter interactions with the tyrosine kinase inhibitors imatinib, nilotinib, and dasatinib. Drug Metab. Dispos..

[31] Giles F. J., Kantarjian H. M., Cortes J., Thomas D. A., Talpaz M., Manshouri T., Albitar M. (1999). Multidrug resistance protein expression in chronic myeloid leukemia: associations and significance. Cancer.

[32] Vasconcelos F. C., Silva K. L., De Souza P. S., Silva L. F. R., Moellmann-Coelho A., Klumb C. E., Maia R. C. (2011). Variation of MDR proteins expression and activity levels according to clinical status and evolution of CML patients. Cytometry B.

[33] Vasconcelos F. C., Cavalcanti G. B., Silva K. L., de Meis E., Kwee J. K., Rumjanek V. M., Maia R. C. (2007). Contrasting features of MDR phenotype in leukemias by using two fluorochromes: implications for clinical practice. Leuk. Res..

[34] Burger H., van Tol H., Brok M., Wiemer E. A. C., de Bruijn E. A., Guetens G., de Boeck G., Sparreboom A., Verweij J., Nooter K. (2005). Chronic imatinib mesylate exposure leads to reduced intracellular drug accumulation by induction of the ABCG2 (BCRP) and ABCB1 (MDR1) drug transport pumps. Cancer Biol. Ther..

[35] Stromskaya T. P., Rybalkina E. Y., Kruglov S. S., Zabotina T. N., Mechetner E. B., Turkina A. G., Stavrovskaya A. A. (2008). Role of P-glycoprotein in evolution of populations of chronic myeloid leukemia cells treated with imatinib. Biochemistry (Mosc).

[36] Hait W. N., Choudhury S., Srimatkandada S., Murren J. R. (1993). Sensitivity of K562 human chronic myelogenous leukemia blast cells transfected with a human multidrug resistance cDNA to cytotoxic drugs and differentiating agents. J. Clin. Invest..

[37] Reis F. R. S., Vasconcelos F. C., Pereira D. L., Moellman-Coelho A., Silva K. L., Maia R. C. (2011). Survivin and P-glycoprotein are associated and highly expressed in late phase chronic myeloid leukemia. Oncol. Rep..

[38] Grandjean F., Brémaud L., Verdier M., Robert J., Ratinaud M. H. (2001). Sequential gene expression of P-glycoprotein (P-gp), multidrug resistance-associated protein (MRP) and lung resistance protein: functional activity of P-gp and MRP present in the doxorubicin-resistant human K562 cell lines. Anticancer Drugs.

[39] Daflon-Yunes N., Pinto-Silva F. E., Vidal R. S., Novis B. F., Berguetti T., Lopes R. R. S., Polycarpo C., Rumjanek V. M. (2013). Characterization of a multidrug resistant chronic myeloid leukemia cell line presenting multiple resistance mechanisms. Mol. Cell. Biochem..

[40] Lopes E. C., Ernst G., Aulicino P., Vanzulli S., García M., Alvarez E., Hajos S. E. (2002). Dissimilar invasive and metastatic behavior of vincristine and doxorubicin-resistant cell lines derived from a murine T cell lymphoid leukemia. Clin. Exp. Metastasis.

[41] Rumjanek V. M., Trindade G. S., Wagner-Souza K., de-Oliveira M. C., Marques-Santos L. F., Maia R. C., Capella M. A. (2001). Multidrug resistance in tumour cells: characterization of the multidrug resistant cell line K562-Lucena 1. Anais da Academia Brasileira de Ciências.

[42] Mahon F.-X., Belloc F., Lagarde V., Chollet C., Moreau-Gaudry F., Reiffers J., Goldman J. M., Melo J. V. (2003). MDR1 gene overexpression confers resistance to imatinib mesylate in leukemia cell line models. Blood.

[43] Illmer T., Schaich M., Platzbecker U., Freiberg-Richter J., Oelschlägel U., von Bonin M., Pursche S., Bergemann T., Ehninger G., Schleyer E. (2004). P-glycoprotein-mediated drug efflux is a resistance mechanism of chronic myelogenous leukemia cells to treatment with imatinib mesylate. Leukemia.

[44] Nakanishi T., Shiozawa K., Hassel B. A., Ross D. D. (2006). Complex interaction of BCRP/ABCG2 and imatinib in BCR–ABL-expressing cells: BCRP-mediated resistance to imatinib is attenuated by imatinib-induced reduction of BCRP expression. Blood.

[45] Tsuruo T., Iida H., Tsukagoshi S., Sakurai Y. (1981). Overcoming of vincristine resistance in P388 leukemia *in vivo* and *in vitro* through enhanced cytotoxicity of vincristine and vinblastine by verapamil. Cancer Res..

[46] Cumber P. M., Jacobs A., Hoy T., Whittaker J. A., Tsuruo T., Padua R. A. (1991). Increased drug accumulation *ex vivo* with cyclosporin in chronic lymphatic leukemia and its relationship to epitope masking of P-glycoprotein. Leukemia.

[47] Wu C.-P., Calcagno A. M., Ambudkar S. V. (2008). Reversal of ABC drug transporter-mediated multidrug resistance in cancer cells: evaluation of current strategies. Curr. Mol. Pharmacol..

[48] Brózik A., Hegedüs C., Erdei Z., Hegedus T., Özvegy-Laczka C., Szakács G., Sarkadi B. (2011). Tyrosine kinase inhibitors as modulators of ATP binding cassette multidrug transporters: substrates, chemosensitizers or inducers of acquired multidrug resistance?. Expert Opin. Drug Metab. Toxicol..

[49] List A. F., Kopecky K. J., Willman C. L., Head D. R., Slovak M. L., Douer D., Dakhil S. R., Appelbaum F. R. (2002). Cyclosporine inhibition of P-glycoprotein in chronic myeloid leukemia blast phase. Blood.

[50] Maia R. C., Noronha H., Vasconcelos F. C., Rumjanek V. M. (1997). Interaction of cyclosporin A and etoposide. Clinical and *in vitro* assessment in blast phase of chronic myeloid leukaemia. Clin. Lab. Haematol..

[51] Maia R. C., Carriço M. K., Klumb C. E., Noronha H., Coelho A. M., Vasconcelos F. C., Ruimanek V. M. (1997). Clinical approach to circumvention of multidrug resistance in refractory leukemic patients: association of cyclosporin A with etoposide. J. Exp. Clin. Cancer Res..

[52] Palmeira A., Sousa E., Vasconcelos M. H., Pinto M. M. (2012). Three decades of P-gp inhibitors: skimming through several generations and scaffolds. Curr. Med. Chem..

[53] Honda H., Ushijima T., Wakazono K., Oda H., Tanaka Y., Aizawa S. I, Ishikawa T., Yazaki Y., Hirai H. (2000). Acquired loss of p53 induces blastic transformation in p210(bcr/abl)-expressing hematopoietic cells: a transgenic study for blast crisis of human CML. Blood.

[54] Di Bacco A., Keeshan K., McKenna S. L., Cotter T. G. (2000). Molecular abnormalities in chronic myeloid leukemia: deregulation of cell growth and apoptosis. Oncologist.

[55] Bi S., Lanza F., Goldman J. M. (1994). The involvement of ‘tumor suppressor’ p53 in normal and chronic myelogenous leukemia hemopoiesis. Cancer Res..

[56] Ashur-Fabian O., Adamsky K., Trakhtenbrot L., Cohen Y., Raanani P., Hardan I., Nagler A., Rechavi G., Amariglio N. (2007). Apaf1 in chronic myelogenous leukemia (CML) progression: reduced Apaf1 expression is correlated with a H179R p53 mutation during clinical blast crisis. Cell Cycle.

[57] Cavalcanti G. B., Scheiner M. A. M., Simões Magluta E. P., Vasconcelos F. D. C., Klumb C. E., Maia R. C. (2010). P53 flow cytometry evaluation in leukemias: correlation to factors affecting clinical outcome. Cytometry B.

[58] Bi S., Hughes T., Bungey J., Chase A., de Fabritiis P., Goldman J. M. (1992). p53 in chronic myeloid leukemia cell lines. Leukemia.

[59] Bedi A., Zehnbauer B. A., Barber J. P., Sharkis S. J., Jones R. J. (1994). Inhibition of apoptosis by BCR-ABL in chronic myeloid leukemia. Blood.

[60] Amarante-Mendes G. P., McGahon A. J., Nishioka W. K., Afar D. E., Witte O. N., Green D. R. (1998). Bcl-2-independent Bcr-Abl-mediated resistance to apoptosis: protection is correlated with up regulation of Bcl-xL. Oncogene.

[61] Gesbert F., Griffin J. D. (2000). Bcr/Abl activates transcription of the Bcl-X gene through STAT5. Blood.

[62] Ravandi F., Kantarjian H. M., Talpaz M., O’Brien S., Faderl S., Giles F. J., Thomas D., Cortes J., Andreeff M., Estrov Z. (2001). Expression of apoptosis proteins in chronic myelogenous leukemia: associations and significance. Cancer.

[63] Aichberger K. J., Mayerhofer M., Krauth M.-T., Skvara H., Florian S., Sonneck K., Akgul C., Derdak S., Pickl W. F., Wacheck V. (2005). Identification of mcl-1 as a BCR/ABL-dependent target in chronic myeloid leukemia (CML): evidence for cooperative antileukemic effects of imatinib and mcl-1 antisense oligonucleotides. Blood.

[64] Deming P. B., Schafer Z. T., Tashker J. S., Potts M. B., Deshmukh M., Kornbluth S. (2004). Bcr-Abl-mediated protection from apoptosis downstream of mitochondrial cytochrome c release. Mol. Cell. Biol..

[65] Gonzalez M. S., De Brasi C. D., Bianchini M., Gargallo P., Moiraghi B., Bengió R., Larripa I. B. (2010). BAX/BCL-XL gene expression ratio inversely correlates with disease progression in chronic myeloid leukemia. Blood Cells Mol. Dis..

[66] Aichberger K. J., Mayerhofer M., Krauth M.-T., Vales A., Kondo R., Derdak S., Pickl W. F., Selzer E., Deininger M., Druker B. J. (2005). Low-level expression of proapoptotic Bcl-2-interacting mediator in leukemic cells in patients with chronic myeloid leukemia: role of BCR/ABL, characterization of underlying signaling pathways, and reexpression by novel pharmacologic compounds. Cancer Res..

[67] Kuribara R., Honda H., Matsui H., Shinjyo T., Inukai T., Sugita K., Nakazawa S., Hirai H., Ozawa K., Inaba T. (2004). Roles of Bim in apoptosis of normal and Bcr–Abl-expressing hematopoietic progenitors. Mol. Cell. Biol..

[68] Wagner-Souza K., Echevarria-Lima J., Rodrigues L. A. P., Reis M., Rumjanek V. M. (2003). Resistance to thapsigargin-induced intracellular calcium mobilization in a multidrug resistant tumour cell line. Mol. Cell. Biochem..

[69] Selleri C., Maciejewski J. P., Pane F., Luciano L., Raiola A. M., Mostarda I., Salvatore F., Rotoli B. (1998). Fas-mediated modulation of Bcr/Abl in chronic myelogenous leukemia results in differential effects on apoptosis. Blood.

[70] Selleri C., Maciejewski J. P. (2000). The role of FAS-mediated apoptosis in chronic myelogenous leukemia. Leuk. Lymph..

[71] Greaney P., Nahimana A., Lagopoulos L., Etter A.-L., Aubry D., Attinger A., Beltraminelli N., Huni B., Bassi I., Sordat B. (2006). A Fas agonist induces high levels of apoptosis in haematological malignancies. Leuk. Res..

[72] Peter M. E., Legembre P., Barnhart B. C. (2005). Does CD95 have tumor promoting activities?. Biochim. Biophys. Acta.

[73] Legembre P., Barnhart B. C., Zheng L., Vijayan S., Straus S. E., Puck J., Dale J. K., Lenardo M., Peter M. E. (2004). Induction of apoptosis and activation of NF-kappaB by CD95 require different signalling thresholds. EMBO Rep..

[74] McGahon A. J., Nishioka W. K., Martin S. J., Mahboubi A., Cotter T. G., Green D. R. (1995). Regulation of the Fas apoptotic cell death pathway by Abl. J. Biol. Chem..

[75] Munker R., Marini F., Jiang S., Savary C., Owen-Schaub L., Andreeff M. (1997). Expression of CD95(FAS) by gene transfer does not sensitize K562 to Fas-killing. Hematol. Cell Ther..

[76] Belloc F., Cotteret S., Labroille G., Schmit V., Jaloustre C., Dumain P., Durrieu F., Reiffers J., Boisseau M. R., Bernard P. (1997). Bcr–abl translocation can occur during the induction of multidrug resistance and confers apoptosis resistance on myeloid leukemic cell lines. Cell Death Differ..

[77] Fulda S., Vucic D. (2012). Targeting IAP proteins for therapeutic intervention in cancer. Nature Rev. Drug Disc..

[78] Li F., Ambrosini G., Chu E. Y., Plescia J., Tognin S., Marchisio P. C., Altieri D. C. (1998). Control of apoptosis and mitotic spindle checkpoint by survivin. Nature.

[79] Hernández-Boluda J.-C., Bellosillo B., Vela M.-C., Colomer D., Alvarez-Larrán A., Cervantes F. (2005). Survivin expression in the progression of chronic myeloid leukemia: a sequential study in 16 patients. Leuk. Lymph..

[80] Conte E., Stagno F., Guglielmo P., Scuto A., Consoli C., Messina A. (2005). Survivin expression in chronic myeloid leukemia. Cancer Lett..

[81] Johnstone R. W., Ruefli A. A., Tainton K. M., Smyth M. J. (2000). A role for P-glycoprotein in regulating cell death. Leuk. Lymph..

[82] Tamm I., Richter S., Oltersdorf D., Creutzig U., Harbott J., Scholz F., Karawajew L., Ludwig W.-D., Wuchter C. (2004). High expression levels of x-linked inhibitor of apoptosis protein and survivin correlate with poor overall survival in childhood *de novo* acute myeloid leukemia. Clin. Cancer Res..

[83] Silva K. L., de Souza P. S., de Moraes G. N., Moellmann-Coelho A., da Cunha Vasconcelos F., Maia R. C. (2013). XIAP and P-glycoprotein co-expression is related to imatinib resistance in chronic myeloid leukemia cells. Leuk. Res..

[84] Hu M., Liu Y., Deng C., Han R., Jia Y., Liu S., Jiang Z., Cao X., He L., Zhang Q. (2011). Enhanced invasiveness in multidrug resistant leukemic cells is associated with overexpression of P-glycoprotein and cellular inhibitor of apoptosis protein. Leuk. Lymph..

[85] Souza P. S., Vasconcelos F. C., De Souza Reis F. R., Nestal De Moraes G., Maia R. C. (2011). P-glycoprotein and survivin simultaneously regulate vincristine-induced apoptosis in chronic myeloid leukemia cells. Int. J. Oncol..

[86] Bernardo P. S., de Souza Reis F. R., Maia R. C. (2012). Imatinib increases apoptosis index through modulation of survivin subcellular localization in the blast phase of CML cells. Leuk. Res..

[87] Quintás-Cardama A., Kantarjian H., Garcia-Manero G., O’Brien S., Faderl S., Ravandi F., Giles F., Thomas D., Wierda W., Cortes J. (2007). A pilot study of imatinib, low-dose cytarabine and idarubicin for patients with chronic myeloid leukemia in myeloid blast phase. Leuk. Lymph..

[88] Ciarcia R., d’Angelo D., Pacilio C., Pagnini D., Galdiero M., Fiorito F., Damiano S., Mattioli E., Lucchetti C., Florio S. (2010). Dysregulated calcium homeostasis and oxidative stress in chronic myeloid leukemia (CML) cells. J. Cell. Physiol..

[89] Sulová Z., Seres M., Barancík M., Gibalová L., Uhrík B., Poleková L., Breier A. (2009). Does any relationship exist between P-glycoprotein-mediated multidrug resistance and intracellular calcium homeostasis. Gen. Physiol. Biophys..

[90] Breier A., Gibalova L., Seres M., Barancik M., Sulova Z. (2013). New insight into p-glycoprotein as a drug target. Anti-cancer Agents Med. Chem..

[91] Bernardo A. A., Pinto-Silva F. E., Persechini P. M., Coutinho-Silva R., Meyer-Fernandes J. R., de Souza A. L. F., Rumjanek V. M. (2006). Effect of extracellular ATP on the human leukaemic cell line K562 and its multidrug counterpart. Mol. Cell. Biochem..

[92] Jamieson C. H. (2008). Chronic myeloid leukemia stem cells. Hematology/the Education Program of the American Society of Hematology. American Society of Hematology. Education Program.

[93] Tang L., Bergevoet S. M., Gilissen C., de Witte T., Jansen J. H., van der Reijden B. A., Raymakers R. A. P. (2010). Hematopoietic stem cells exhibit a specific ABC transporter gene expression profile clearly distinct from other stem cells. BMC Pharmacol..

[94] Porro A., Iraci N., Soverini S., Diolaiti D., Gherardi S., Terragna C., Durante S., Valli E., Kalebic T., Bernardoni R. (2011). c-MYC oncoprotein dictates transcriptional profiles of ATP-binding cassette transporter genes in chronic myelogenous leukemia CD^34+^ hematopoietic progenitor cells. Mol. Cancer Res..

[95] Naka K., Hoshii T., Hirao A. (2010). Novel therapeutic approach to eradicate tyrosine kinase inhibitor resistant chronic myeloid leukemia stem cells. Cancer Sci..

[96] Sengupta A., Banerjee D., Chandra S., Banerji S. K., Ghosh R., Roy R., Banerjee S. (2007). Deregulation and cross talk among Sonic hedgehog, Wnt, Hox and Notch signaling in chronic myeloid leukemia progression. Leukemia.

[97] Queiroz K. C. S., Ruela-de-Sousa R. R., Fuhler G. M., Aberson H. L., Ferreira C. V, Peppelenbosch M. P., Spek C. A. (2010). Hedgehog signaling maintains chemoresistance in myeloid leukemic cells. Oncogene.

[98] Corrêa S., Binato R., Du Rocher B., Castelo-Branco M. T. L., Pizzatti L., Abdelhay E. (2012). Wnt/β-catenin pathway regulates ABCB1 transcription in chronic myeloid leukemia. BMC Cancer.

[99] Marques D. S., Sandrini J. Z., Boyle R. T., Marins L. F., Trindade G. S. (2010). Relationships between multidrug resistance (MDR) and stem cell markers in human chronic myeloid leukemia cell lines. Leuk. Res..

[100] Xin H., Kong Y., Jiang X., Wang K., Qin X., Miao Z.-H., Zhu Y., Tan W. (2013). Multi-drug-resistant cells enriched from chronic myeloid leukemia cells by Doxorubicin possess tumor-initiating-cell properties. J. Pharmacol. Sci..

[101] Wang X. Q., Ongkeko W. M., Chen L., Yang Z. F., Lu P., Chen K. K., Lopez J. P., Poon R. T. P., Fan S. T. (2010). Octamer 4 (Oct4) mediates chemotherapeutic drug resistance in liver cancer cells through a potential Oct4-AKT-ATP-binding cassette G2 pathway. Hepatology.

[102] Hochedlinger K., Yamada Y., Beard C., Jaenisch R. (2005). Ectopic expression of Oct-4 blocks progenitor-cell differentiation and causes dysplasia in epithelial tissues. Cell.

[103] Preisinger C., Kolch W. (2010). The Bcr-Abl kinase regulates the actin cytoskeleton via a GADS/Slp-76/Nck1 adaptor protein pathway. Cell. Signal..

[104] Erokhina M. V, Stavrovskaya A. A., Onishchenko G. E. (1999). Golgi complex is brefeldin A resistant in multidrug resistant cells. Membr. Cell Biol..

[105] De Souza Votto A. P., Renon V. P., Yunes J. S., Rumjanek V. M., Marques Capella M. A., Neto V. M., Sampaio de Freitas M., Alicia Geracitano L., Monserrat J. M., Trindade G. S. (2007). Sensitivity to microcystins: a comparative study in human cell lines with and without multidrug resistance phenotype. Cell Biol. Int..

[106] Ferreira P. a., Ruela-de-Sousa R. R., Queiroz K. C. S., Souza A. C. S., Milani R., Pilli R. A., Peppelenbosch M. P., den Hertog J., Ferreira C. V. (2012). Knocking down low molecular weight protein tyrosine phosphatase (LMW-PTP) reverts chemoresistance through inactivation of Src and Bcr-Abl proteins. PloS ONE.

[107] Vasconcelos F. C., Gattass C. R., Rumjanek V. M., Maia R. C. (2007). Pomolic acid-induced apoptosis in cells from patients with chronic myeloid leukemia exhibiting different drug resistance profile. Invest. New Drugs.

[108] Maia R. C., Vasconcelos F. C., de Sá Bacelar T., Salustiano E. J., da Silva L. F. R., Pereira D. L., Moellman-Coelho A., Netto C. D., da Silva A. J., Rumjanek V. M. (2011). LQB-118, a pterocarpanquinone structurally related to lapachol [2-hydroxy-3-(3-methyl-2-butenyl)-1,4-naphthoquinone]: a novel class of agent with high apoptotic effect in chronic myeloid leukemia cells. Invest. New Drugs.

[109] Trindade G. S., Capella M. A., Capella L. S., Affonso-Mitidieri O. R., Rumjanek V. M. (1999). Differences in sensitivity to UVC, UVB and UVA radiation of a multidrug-resistant cell line overexpressing P-glycoprotein. Photochem. Photobiol..

[110] Dias M. C., Votto A. P., Filgueira D. D., Almeida D. V., Vallochi A. L., D Oca M. G., Marins L. F., Trindade G. S. (2011). Anti-MDR and antitumoral action of acetylsalicylic acid on leukemic cells. Biosci. Rep..

[111] Fernandes J., Castilho R. O., da Costa M. R., Wagner-Souza K., Coelho Kaplan M. A., Gattass C. R. (2003). Pentacyclic triterpenes from Chrysobalanaceae species: cytotoxicity on multidrug resistant and sensitive leukemia cell lines. Cancer Lett..

[112] da Rocha G. G., Simões M., Lúcio K. A., Oliveira R. R., Coelho Kaplan M. A., Gattass C. R. (2007). Natural triterpenoids from Cecropia lyratiloba are cytotoxic to both sensitive and multidrug resistant leukemia cell lines. Bioorg. Med. Chem..

[113] Capella M. A. M., Capella L. S., Valente R. C., Gefé M., Lopes A. G. (2007). Vanadate-induced cell death is dissociated from H2O2 generation. Cell Biol. Toxicol..

[114] Kirszberg C., Rumjanek V. M., Capella M. A. M. (2005). Methylene blue is more toxic to erythroleukemic cells than to normal peripheral blood mononuclear cells: a possible use in chemotherapy. Cancer Chemother. Pharmacol..

[115] Silva K. L., Vasconcelos F. C., Marques-Santos L. F., Kwee J. K., Maia R. C. (2003). CPT-11-induced cell death in leukemic cells is not affected by the MDR phenotype. Leuk. Res..

[116] Netto C. D., Santos E. S. J., Castro C. P., da Silva A. J. M., Rumjanek V. M., Costa P. R. R. (2009). (+/−)-3,4-Dihydroxy-8,9-methylenedioxypterocarpan and derivatives: cytotoxic effect on human leukemia cell lines. Eur. J. Med. Chem..

[117] Salustiano E. J. S., Netto C. D., Fernandes R. F., da Silva A. J. M., Bacelar T. S., Castro C. P., Buarque C. D., Maia R. C., Rumjanek V. M., Costa P. R. R. (2010). Comparison of the cytotoxic effect of lapachol, alpha-lapachone and pentacyclic 1,4-naphthoquinones on human leukemic cells. Invest. New Drugs.

[118] Netto C. D., da Silva A. J. M., Salustiano E. J. S., Bacelar T. S., Riça I. G., Cavalcante M. C. M., Rumjanek V. M., Costa P. R. R. (2010). New pterocarpanquinones: synthesis, antineoplasic activity on cultured human malignant cell lines and TNF-alpha modulation in human PBMC cells. Bioorg. Med. Chem..

[119] Kanehisa M., Goto S., Sato Y., Furumichi M., Tanabe M. (2012). KEGG for integration and interpretation of large-scale molecular data sets. Nucleic Acids Res..

[120] Scotto K. W. (2003). Transcriptional regulation of ABC drug transporters. Oncogene.

